# Making seafood accessible to low-income and nutritionally vulnerable populations on the U.S. West Coast

**DOI:** 10.5304/jafscd.2020.101.027

**Published:** 2020-12-11

**Authors:** J. Zachary Koehn, Emilee L. Quinn, Jennifer J. Otten, Edward H. Allison, Christopher M. Anderson

**Affiliations:** a School of Aquatic & Fishery Sciences, College of the Environment, University of Washington; 1122 NE Boat Street, Box 355020; Seattle, WA 98195 USA;; b Center for Ocean Solutions, Stanford University; 473 Via Ortega; Stanford, CA USA,; c Center for Public Health Nutrition, Department of Health Services, School of Public Health, University of Washington; Raitt Hall; Seattle, WA 98105 USA;; d Nutritional Sciences Program, Center for Public Health Nutrition, Department of Environmental and Occupational Health Sciences, School of Public Health, University of Washington; Raitt Hall; Seattle, WA 98105 USA;; e Nippon Foundation Ocean Nexus Program, Earthlab, University of Washington; 3707 Brooklyn Ave. NE; Seattle, WA 98105 USA;; f WorldFish, Jalan Batu Maung, Batu Maung, Bayan Lepas, Penang, Malaysia.; g School of Aquatic & Fishery Sciences, College of the Environment, University of Washington; 1122 NE Boat Street, Box 355020; Seattle, WA 98195 USA;

**Keywords:** Fisheries, Food System, Seafood, Local Food, Food Access, Health, Low-income Populations

## Abstract

Along the U.S. West Coast, sustainable management has rebuilt fish stocks, providing an opportunity to supply nutrient-rich food to adjacent coastal communities where food insecurity and diet-based diseases are common. However, the market has not successfully supplied locally sourced seafood to nutritionally vulnerable people. Rather, a few organizations make this connection on a limited scale. We used a “positive deviant” approach to learn how these organizations’ efforts developed, how they overcame challenges, and what conditions enabled their interventions. We found that organizations in these positive deviant cases provided fish from a wide variety of species and sources, and distributed them through different channels to a diversity of end consumers. A key factor facilitating success was the ability to negotiate a price point that was both profitable and reasonable for organizations supplying nutritionally vulnerable or low-income consumers. Further-more, securing access to grants overcame initial costs of establishing new supply channels. All cases highlighted the importance of individual champions who encouraged development and cultural connections between the initiative and the nearby community. Organizations overcame key challenges by establishing regulations governing these new channels and either using partnerships or vertically integrating to reduce costs associated with processing and transport. Oftentimes training and education were also critical to instruct workers on how to process unfamiliar fish and to increase consumer awareness of local fish and how to prepare them. These lessons illuminate pathways to improve the contribution of local seafood to the healthy food system.

## Introduction

In much of the world, overfishing and the consequent need to restrict fishing levels to sustain stocks is a key issue affecting people’s access to fish as a nutritious food. This is not a problem on the West Coast of the United States, where almost all commercially harvested fish populations are now abundant enough to be classified as rebuilt due to science-based and conservation-focused management (National Oceanic and Atmospheric Adminstration Fisheries, 2019). Despite this, harvests for many species remain far below what biologists advise as sustainable, in part due to low demand for some abundant fish species, which are known as “underutilized” species.

These abundant and low-cost fishery resources exist alongside human populations that could benefit from affordable, culturally appropriate, and healthy food options. These include coastal tribes, populations traditionally reliant on seafood, as well as economically disadvantaged communities—some of which are or once were centered around industrial fishing (Sepez et al., 2007). The food environment along the U.S. West Coast reflects a familiar problem where an available source of healthy food—in this case underutilized local fish—is inaccessible to low-income and food insecure people in rural communities located near this significant food source (Larson, Story, & Nelson, 2009; Shannon, Kim, McKenzie, & Lawrence, 2015).

### Seafood Consumption in the United States

Across the United States, seafood consumption is lower than recommended levels: between 80–90% of Americans who eat fish consume only one fifth to one half of the recommended weekly intake of 2 servings (Jahns et al., 2014; U.S. Department of Health and Human Services & U.S. Department of Agriculture [USDA], 2015), including along the West Coast (U.S. Environmental Protection Agency, 2014). Further, the amount of seafood individuals eat differs by age and social class: younger or lower-income populations eat less seafood than older and higher-income people (Jahns et al., 2014; Love, Asche et al., 2020). This disparity is not recent; a 1995 survey found that low-income pregnant mothers ate about half the recommended intake of fish needed for Omega-3 polyunsaturated fatty acids (PUFAs), which are critical to maternal and early childhood development (Lewis, Widga, Buck, & Frederick, 1996). Nutrition surveys found that the U.S. population generally has low concentrations of two PUFAs derived from marine-sourced foods—docosahex-aenoic and eicosapentaenoic acid (DHA and EPA)—and rates of intake differ by race and ethnicity (U.S. Center for Disease Control & National Center for Environmental Health, 2012). For example, non-Hispanic Blacks and whites had higher concentrations of EPA than Mexican American adults (U.S. Center for Disease Control & National Center for Environmental Health, 2014). While there is limited publicly available data on consumer taste preferences for fish and price-related choices, existing data highlights consumers’ lack of understanding about how to prepare and handle seafood alongside the perception that seafood is more expensive than other animal-based foods (Jahns et al., 2014).

Increasing the consumption of nutrient-rich fish can aid in addressing nutrient deficiencies that are prevalent in the U.S. The Dietary Guidelines for Americans notes that potassium, dietary fiber, choline, magnesium, calcium, and Vitamins A, D, E, and C are under-consumed; of these, calcium, potassium, dietary fiber, and Vitamin D are considered “nutrients of public health concern” because low intakes are associated with poorer health outcomes (U.S. Department of Health and Human Services & USDA, 2015). Several of these micronutrients are present in high concentrations (e.g., Vitamin A and calcium, Vitamin B12, iron, zinc) and in more bioavailable forms in fish and shellfish than they are in many vegetables, fortified staples, and food supplements (Bogard et al., 2015). Increasing consumption of local, sustainable fish in poorer communities on the West Coast is one pathway toward improving diet-related health outcomes. Fish and shellfish could contribute a nutrient-rich source of food if they were made more available and accessible.

### Capture Fisheries on the U.S. West Coast and the Seafood Supply Chain

Seafood availability and accessibility is influenced significantly by supply chains, including the characteristics and management of large fisheries focused on particular species and jurisdictions. Fisheries along the West Coast vary considerably, from large volume fisheries targeting single species destined for export to small scale operators who target multiple species selling to metropolitan centers and restaurants. Despite this diversity, seafood products tend to follow a similar pathway through the supply chain (see Box 1 for a description of the traditional seafood supply chain).

The fishery predominantly in focus here is the West Coast groundfish, a complex fishery that harvests over 100 species in a variety of sea-bed habi-tats using multiple fishing gears. This fishery is guided by a science-based management plan over-seen by the federal government and is increasingly considered a sustainability success story. However, it has not always been that way.

Landings of groundfish species on the West Coast increased through the 1970s, particularly by foreign vessels. The UN Convention on the Law of the Sea established exclusive economic zones along the coastline and by 1976 the U.S. passed federal laws that excluded foreign vessels from federal waters. Over the next decade, the domestic fleet saw high production, and new vessels were drawn to the fishery, leading to overfishing. By the late 1990s, many of the species were in rapid decline, and overcapitalization combined with resulting labor losses prompted the U.S. Secretary of Commerce, in 2000, to declare the West Coast groundfish fishery an official national disaster. Over the next decade, science-based and conservation-focused management restricted fishing harvests while stocks were carefully observed, and fish populations rebuilt. Now, almost all commercially important fish populations in the groundfish fishery are once again abundant enough to be classified as rebuilt by fishery managers.

Each year, the Pacific Fishery Management Council approves maximum total allowable catch (TAC) levels that ensure harvests are sustainable for federal fisheries along the continental West Coast. Harvest rates have remained far below these levels for most species; TAC is reached only for high value species with demand in regional or international markets (e.g., Sablefish, *Anoplopoma fimbria*, for export and Petrale Sole, *Eopsetta jordani*, for domestic urban markets). For the majority of groundfish species that lack markets, continued low catch means inconsistent supply for processors and local markets which, in turn, perpetuates low demand. In the years following the collapse, processors and wholesalers were forced to switch to “less discriminating protein markets” to stay in business—presumably switching from the overfished species to more consistently affordable and available imported and farmed fish like tilapia (may include *Coptodon sp., Oreochormis sp*., and *Sarotherodon sp*.) and Alaskan pollock (*Gadus chalcogrammus*) (Errend, Gilden, Harley, Morrison, Pfeiffer, Russell, & Seger, 2017).

Underutilized species provide an opportunity to sustainably supply affordable micronutrients for West Coast consumers. Underutilized species may be those that have weak markets and are therefore undesirable or they may include bycatch—fish caught unintentionally while targeting another species. While the global issue of bycatch remains a challenge in the U.S., U.S. federal fisheries are required by law to establish monitoring programs, to adhere to protected species programs, and to minimize bycatch to the extent practicable. A recent assessment indicated that current rates of U.S. bycatch have declined, especially on the West Coast and Alaska (Savoca et al., 2020). Bycatch rates also vary by the type of fishing gear used. The sources of fish in this study originate primarily from trawl-type fishing gears, which involves trawling a net over the seabed. Trawl fishing bycatch in the U.S. was found to be much lower than in much of the world, due in part to strong management (Pérez-Roda et al., 2019; Savoca et al., 2020). The results of relying on media messages focused on consumption of underutilized species are mixed. On the other hand, increasing the availability and diversity of underutilized species that originate from well managed fisheries gives consumers greater options and variety of sustainably harvested species (Farmery, van Putten, Phillipov, & McIlgorm, 2020).

Few analyses have evaluated how local seafood production may contribute to food insecurity and malnutrition in low-income communities residing in more developed country contexts such as the West Coast. In the Kenai region of Alaska, local seafood harvest supported rural livelihoods and nutrition for low-income households; they encouraged creation of more local markets for seafood to further strengthen coastal community food systems (Loring, Gerlach, & Harrison, 2013). In Southern California, access to seafood markets decreased mere kilometers inland from the coast, and even when seafood markets are present in these areas, local seafood is not often sold (Talley, Warde, & Venuti, 2016). To date, research in developed country contexts has not focused on initiatives that actively seek to supply seafood to low-income and nutritionally vulnerable communities.

### Study Purpose

With evidence of low consumption of fish along the West Coast and increasingly abundant capture fisheries offshore, there are emerging opportunities to use seafood to help improve public health and nutrition. The goal of this study was to understand these opportunities more deeply through:

1. Identifying supply-chain actors that have successfully supplied nutritionally vulnerable consumers with affordable, available seafood; identifying how these actors and organizations intervened in the existing seafood supply chain and food system, and
2. Ascertaining the attributes and conditions facilitating their success as well as their strategies for overcoming challenges.

## Methodology

To achieve these goals, we drew on our personal and professional networks as well as on media reports and internet searches to identify organizations, programs, and initiatives along the West Coast that are successfully connecting low-income and nutritionally vulnerable populations with local and underutilized fish (i.e., “positive deviant cases”). We conducted semistructured interviews with actors directly involved in each effort and used a multiple case study approach and qualitative data collection and analysis techniques to describe, compare, and contrast key elements of the case examples. For each case, we aimed to elucidate how they operated within the dominant supply chain(s), how they created new supply chains, the enabling conditions for their success, and how they were able to overcome challenges associated with the distribution of landed fish.

### Positive Deviance Approach

Positive deviance seeks to learn from the individuals or organizations who achieve success where the majority do not (Pascale & Sternin, 2005). In this case, positive deviant case studies have created interventions in seafood markets to supply low-cost but nutritious fish to the nutrition-poor communities of potential fish-eaters, while the majority of the seafood sector have not been able to do this. This juxtaposition of rich fishery resources with undernourished people is a global problem (Hicks et al., 2019); thus, understanding how it might be overcome is of interest beyond the West Coast.

Positive deviance analysis is similar to best practice case studies in that both seek to learn from success. However, positive deviance tends to focus on learning from communities that have found their own solutions rather than on transferring lessons from an external authority. Applying and transmitting knowledge using a positive deviance approach can help communities identify the practices used by successful actors in neighboring communities to encourage a change in attitudes and facilitate such success within the local context (Pascale & Sternin, 2005). The approach has been used to assess food and nutrition security strategies, for example to determine how—despite a positive correlation between diet cost and nutritional quality—some low-income households were able to sustain healthier diets without accruing more cost when prioritizing items for their nutrient quality (Marty et al., 2015). Research focusing on success encourages optimism and more effective collaboration and creative solutions for “navigating the interface of science, policy and practice” (Cvitanovic & Hobday, 2018, p. 4). Positive deviance not only empowers communities to recognize the potential for change in their own community, it also transforms the dialogue towards optimism, catalyzing collaboration and action. Further, it emphasizes the agency within communities, rather than just the need for change.

Based initially on initiatives known to the authors, we compiled a list of organizations that direct underutilized fish to food insecure and nutritionally vulnerable populations using web searches and snowball sampling from initial interviewees. Our main criteria were that the initiative must be actively distributing seafood to low-income consumers and that the lead agency be based on the West Coast. This process resulted in the identification of one nationwide and three local or regional cases (*n*=4). We acquired and used the organizations’ websites, personal contacts, or information from other interviewees to identify contact information for one or more representatives to request interviews. For three of the cases, we interviewed multiple people involved in the effort, including multiple staff from the lead agency and/or stakeholders from partnering agencies. Interviews were conducted one-on-one or with multiple interviewees at a time. For the four case studies, we conducted six separate interviews—four in person and two over the phone, with a total of nine interviewees representing a diversity of organizations throughout the seafood supply chain (Table 1).

### Semistructured Interviews

**Figure 1.**
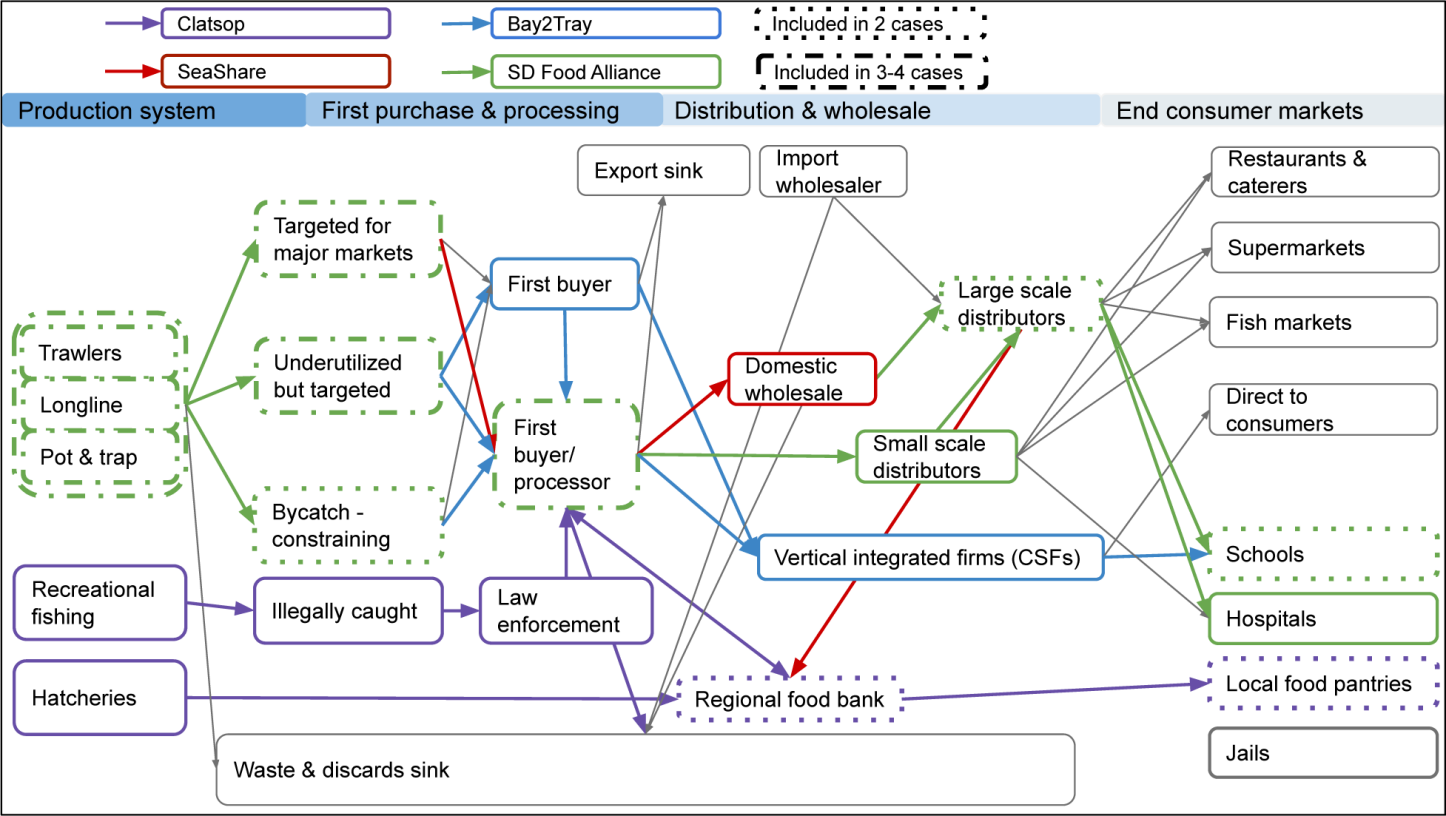
Conceptual Map of Entry Points of Seafood to Vulnerable Populations Used by the Positive Deviant Cases

Semistructured interviews were conducted in-person or via phone. Interview questions related to how the organization’s effort first began and which partners were involved, the fish supply (e.g., fishers and method, amount and type of species caught, marketability and cost); how fish is acquired, processed, and distributed; and descriptions of consumers and how and where they access the fish (e.g., demographics, preferences, food environment). The interview guide also included questions about challenges experienced, factors or conditions that enabled success, and perceived potential for expansion. The interviews were designed to investigate whether and how the cases succeeded or overcame relevant challenges and to illuminate potential opportunities for adapting, replicating, or scaling up any successes. They were also designed to assess our conceptual framework of supply chain pathways for West Coast groundfish (see Figure 1). Interviews lasted up to sixty minutes and were audio recorded with participant permission. The study was approved by the University of Washington Human Subjects Division (IRB ID: STUDY00004939).

### Data Analysis

Interviews were transcribed verbatim and analyzed using Dedoose (version 8.1.8). One team member developed a preliminary code list based on the interview guide. Two team members then separately and independently assigned codes to one transcript, compared their coding, and refined the coding guidance to ensure consistent coding applications. The two team members then coded a second transcript using the updated code list and assessed coding agreement. As only minor adjustments to the code list and definitions were required, one team member coded the remaining transcripts using the refined code list. Team members then reviewed and summarized the transcripts and coded excerpts to identify key elements of each effort (e.g., associated costs, facilitating factors). The team reviewed passages by code and by case to summarize the characteristics and note differences and commonalities across the cases. We then developed a conceptual framework for each case to determine how each either utilized existing market channels in traditional seafood supply chains or created their own channels to link the supply chain. The conceptual framework for each individual positive deviant case study was created separately, and then all case studies were overlaid into a single conceptual framework to visualize similarities and differences in how each of the positive deviants utilized and innovated the traditional seafood supply chain. Finally, we solicited feedback from interviewees on the complete report to ensure that the depiction of each case was accurate.

## Results

First, we present the key characteristics of each of the four cases studied and discuss how these four cases fit into our conceptual framework for a seafood supply chain. Then, we present and describe findings from the interviews on the factors that are particularly important in developing low-cost distribution links for local seafood. These factors are organized into two categories: enabling conditions that facilitated success for the positive deviants and the strategies they used to overcome challenges.

### Key characteristics of the positive deviant case studies

We describe key characteristics of West Coast cases in Table 2.

The positive deviant cases were all nonprofit organizations except for Bay2Tray, which is a program run by a for-profit fish distributor in California. The scale of sourcing and distribution for these initiatives ranged from one or a few adjacent counties to nationwide in the case of SeaShare’s partnership with Feeding America. Seafood sourcing activities varied widely, however most products had lower or no value in standard supply channels. Commercially sourced low-value fish were used across all case studies, but food banks had slightly more regulatory latitude to distribute high value fish from sources that were prohibited to sell it (e.g., fish sourced from illegal harvest). For food banks, commercially sourced fish were donated from various points in the supply chain such as individual boats, surplus inventory from large-scale processors, or from vertically integrated large fishing companies. In some cases, species were profitable fish donated for philanthropic reasons, and in others, donated species were without existing markets. Some referenced species, like Opah (*Lampris guttatus*), are bycaught species caught alongside more desirable target species and have low market value. In delivering fish for schools or larger organizations, some fish were caught on contract. For example, Bay2Tray described working with California fishermen to negotiate price and volume in sourcing fish for its school program.

Less traditional sourcing pathways for food banks also included fish hatcheries and illegally harvested fish seized by law enforcement. Hatchery-raised steelhead and salmon are released to the wild and years later they return to their natal stream—in front of the hatchery—where their eggs and milt are manually harvested; the remaining meat cannot be legally sold but can be donated to food banks. Hatchery returns are variable, but, in some years, millions of fish return and food banks receive hundreds of thousands of kilos of fish (Miller, 2015). Additionally, interviewees highlighted “seized” species apprehended by law enforcement or wild-life officials that were caught in excess of legal limits or were species whose catch is illegal. Enforcement officials contacted food banks as potential outlets for the unsellable, seized fish.

Using a depiction of a typical seafood supply chain as a foundation, we visualized how and where positive deviant interviewees’ efforts might fit within the supply chain (see Box 1). Case study interview data were used to ground truth and adjust our conceptual map of the various pathways by which fish is harvested and reaches consumers (Figure 1).

The four positive deviant cases innovated within the typical supply chain in different ways. In all of these cases, they shortened the supply chain by bundling different aspects of the supply chain or strengthening the relationships with local seafood producers.

### Enabling Conditions that Facilitated Success for Positive Deviants

Interviews pointed towards three specific enabling conditions that facilitated the success of positive deviant cases. Interview coding revealed that details of these conditions varied with the context (Table 3). Each of the cases utilized these enabling conditions to identify gaps and provide the means to bridge the supply chain.

Making connections that supported alternative fish supply chains were critical to establishing markets for large volumes of affordable fish. These connections often depended on, or were deepened by clear mutual benefits and shared values. For example, in connecting local fish to schools, Bay2Tray liaised between fishers, processors, and schools to negotiate products feasible for processors and for school settings, price points, and timing by working as a partnership:

It’s hard work changing the deeply entrenched school food system, so finding a partner who shared values was really important because … when there’s challenges, you both are kind of in it to figure out solutions.

This partnership depended on finding the right balance of affordability for schools and profits for fish businesses. Likewise, in San Diego, the SD Food Systems Alliance saw value in creating local supply chain connections and thus facilitated relationships between regional and local hospitals and schools to more easily purchase local fish. In doing so, they helped fishers gain access to larger, urban markets.

[A fisheries representative and a chef at hospitals] really connected through [the Alliance] and maintaining connections, doing things together, promoting local fishing. For him it’s about the fishermen, for me it’s about health.

In the San Diego case, large institutional buyers (i.e., hospitals, schools) were able to rely on contractual arrangements with broadline distributors who, once notified of the interest in local seafood, were able to source from nearby fishers and smaller distributors with whom they held existing relationships. These relationships also allowed fishers to work with processors to develop and sell incidentally caught Opah in a form that is processed locally into products that are affordable and useful for school meals (e.g., ground Opah chili).

Regional food bank initiatives needed to be creative in how they built relationships between previously unconnected actors in the supply chain. For Clatsop, relationships and communication between the hatchery, law enforcement, the local processing plant, and the regional food bank allowed them to establish a process for ensuring timely delivery of fresh product when fish became available. Finally, SeaShare’s national effort provided a central point of entry to food banks for companies with fish donations and food banks with the capacity to handle the product; intentional relationship-making between willing fishing businesses and regional food banks is core to their mission.

Of the four cases, two aimed to develop a program or arrangement that would eventually prove profitable for fishers and the two others relied on distributing and securing donated fish. In both models, costs were incurred in developing the alternative supply chain that the standard markets were unable to cover. All positive deviant cases described reliance on grants, at least initially, to broadly support program actions or to hire program coordinators. For partnerships between public entities and private enterprise (fishing business) it was stressed that scaling is necessary for the program to become economically viable, and up until that point it was also necessary to find funding from foundations or government. Fishing companies and processors acknowledged the benefit of tax deductions to offset the costs of their donations to food banks. While some large-scale fishing companies were taking advantage of tax deduction incentives, processors in Oregon found that their contributions to the regional food bank consumed so few resources relative to their overall business that they did not see the need to seek donation-based tax deduction. During the three- to four-month period that one large processor prepared fish for food banks, the daily volume of fish processed for donations never exceeded 2,000 to 3,000 pounds (900–1360 kg), compared to their total daily volume ranging from 300,000 to 400,000 pounds (136,000–181,400 kg).

Whatever we can do to help a community and put [our company] in a positive light in our community is what we always strive to do. But other than that, it was just very little hassle for us. It’s just a small thing we could do to help our community. And that was the whole purpose of it. It’s like, is it an inconvenience for us? No, it isn’t at all.

When asked, the afore-referenced interviewee speculated that they could increase donation-based processing by two- to three-fold before they would even begin to consider seeking tax reductions just based on the additional amount of labor.

For each of the four cases there were specific people, or champions, who shouldered the task of identifying and recruiting direct partners and circumventing the established supply channel. For example, these champions recruited people within the fishing industry who were willing to donate processing, storage, and freight (e.g., SeaShare board members, Pacific Seafoods processing company). For schools and hospitals, champions included those in leadership roles (e.g., nutrition directors, superintendents) who decided to innovate with programs like Bay2Tray, or kitchen workers, who were willing to learn to cook with fish from scratch, as stated by two interviewees:

Participant 1: I think a big part of this … something that often gets overlooked, is … leadership in planning a community, or organizations that support that level of leadership. So it’s one thing to have a Board making nutrition directives, it’s another thing to have that Director in a school where the people actually support them to do that, make that an initiative. There’s plenty of schools that wouldn’t support that move for budgetary constraints, for union constraints.

Participant 2: And so it then comes down to the leadership that’s in place, specifically in the nutrition department. If you don’t have a Nutrition Director that’s really willing, really committed to something like this, then it’s not very likely to happen.

It was not only persons in operational authority that were effective champions; in high school leadership councils, students were advocates for Bay2Tray and championed an improved connection between the environment and the food served in their cafeterias. Local media outlets also helped publicize and garner support for such programs. Along with the programs’ internal media campaigns, this outreach expanded the message to other school districts and made them more receptive to new supply chains.

Finally, cultural connections to seafood helped align workers throughout the supply chain with the missions of the positive deviant organizations. In communities with fishing heritage, people were more comfortable with fish processing and preparation. Where Bay2Tray operated, for example, many of the school kitchen workers either had relatives or friends who had worked or were currently working in the fisheries sector. Community fishing culture may facilitate the process of finding fishers or processors who are willing to innovate (e.g., keeping bycatch for use in schools). For communities in close proximity to harbors, processors are more available, transportation is less expensive, and the general population likely has more exposure to seafood and seafood processing. Interviewees working in food banks also discussed particular ethnic groups they serve as having strong connections to seafood in their cultural heritage (e.g., Filipinos, Japanese, Chinese workers) and therefore these communities often exhibit a stronger demand for fish. Cultural connections to local seafood, with respect to ethnicity or to place-based fishing heritage, was a strong enabling theme in the positive deviant cases; sourcing local fish was not only seen as cost-effective, sustainable, and healthier, it was also a way to revitalize the local food economy and support community members engaged in the fishing industry.

### Challenges and How to Overcome Them

Positive deviant cases faced a number of challenges in establishing their initiatives but came up with a variety of strategies to overcome them (Table 4).

A significant challenge reported by interviewees focused on the management of costs and volumes to ensure that the price was low enough for organizations serving end-consumers but sufficient to cover costs or result in profit for fishers and processors. Processing was a major contributor to cost. One strategy used was to select fish that are easier to process in order to yield more salable product per whole fish.

Even when food banks receive fish for free, they may still have to pay for collection, distribution, and processing services or labor. Several programs relied on volunteer or donated labor to save the costs of paying another firm to process or distribute the fish. Early in its development, Clatsop food bank relied on in-house labor from workers who had grown up around fishing, but, as they grew, they sought to partner with a nearby processing facility.

The key is food banks not being processors; it’s them being delivery people. Delivery and distribution people. That’s what we can do well. And that’s the key to this.

Clatsop identified how to scale by partnering with key participants in the conventional supply chain. Similarly, in some cases, transportation costs to the processor and food service or retail settings are covered by partners (e.g., SeaShare relies on regional food banks’ transportation network or on partner fishing companies); in other cases, the organization pays for transport associated costs, such as the truck and the driver’s time, as well as refrigeration. Interviewees indicated that transport becomes a major challenge in rural regions or areas further from the coast, where mainstream distributors might be the only way to improve seafood availability.

[Schools] needed distribution, they needed it dropped at each school site, which to like a small guy would kill you. But if you’re SYSCO, it was like their mainstream distributor, it’s no big deal … that was another hurdle that kept them from wanting to really go full-on with the program… how many school districts out there [would want] our fish if they could access it through their mainstream distributor?

Comments like these indicate that mainstream distributors may be called upon to increase the potential reach of local seafood, especially to areas where there are few alternatives:

Interviewer: “Okay. Are there any other parts of the U.S. that are hard to reach?”

Participant 1: “Just because of logistics there’s more seafood consumption on the coast as you go around the states, so you get those seafood deserts kind of in the center part of the U.S.”

Interviewer: “Meaning there’s less demand for product?”

Participant 2: “Less seafood makes it there and less demand and it costs more to get it … It’s improved over the years. And the food banks are much more sophisticated than they were 10 or 15 years ago. Most all of them have several nutritionists on staff and they’re very interested in seafood and really do want seafood products.”

In San Diego, purchasing organizations successfully applied pressure on their mainstream distributors to source local seafood options instead of imported product.

School food service providers and food banks talked about seafood as a protein source to be compared to other animal source foods, and occasionally referenced a single “protein budget”—or a price per serving of protein that was acceptable within their contexts. In several instances, chicken was the product that acted as a benchmark against which other protein sources (such as fish) were considered. Typically, the price of chicken is lower than that of most seafoods, but interviewees indicated that some underutilized and local species of fish can be cheaper than imported fish or chicken. For example, one bycaught species landed in San Diego is “comparable to what they would do if they were putting the usual chicken nuggets and pizza and the other USDA-kind of supplied process foods” (Interviewee). Another interviewee noted that, particularly for organizations with protein budgets such as hospitals or schools, a creative shift of ingredients can facilitate use of higher priced product (i.e., fish). A pound of protein need not be fish alone; it could be fish and beans.

Positive deviant cases indicated that underutilized fish were unfamiliar to many consumers, potentially creating challenges throughout the supply chain from processing to transportation to preparation. Respondents indicated that new species may require training in institutional settings, particularly in schools where scratch-based cooking is not often used. Interviewees also described the need for education to encourage consumers to become more comfortable with alternatives to the “big three” most consumed species: shrimp, salmon, and tuna. Interviewees in all four cases mentioned education as a complementary activity to their primary focus on making fish available. For example, in San Diego, schoolchildren worked with a local celebrity chef to develop recipes using local seafood. Some of these recipes have been integrated with school meal programs. Bay2Tray utilized taste testing with students to determine their preferences for new fish menu items.

Permitting and regulation created challenges to sourcing local fish and shellfish products that were not specifically aligned with commercial sale. In some cases, modifying the supply chain required policy change. In order for bycatch to be used by regional food banks, SeaShare worked with legislators and stakeholders in the pollock fishery to allow them to collect prohibited species catch (PSC) of salmon and halibut. Bycatch of PSC species all have harvest limits set by federal fisheries managers in the fishery that keep incidental harvest below unsustainable levels, and their sale from operators in the fishery is prohibited by law. Clatsop’s regional food bank gained access to hatchery products or illegally harvested fish after the Oregon state legislature unanimously passed House Bill 4068 in 2012 which enabled seized fish, as well as fish returning to hatcheries, to be donated to regional food banks. It is important to note that the policy changes required for SeaShare and Clatsop CRFB operations *did not* authorize the sale of these species of conservation concern, seized fish, or hatchery caught fish. They only created a donation-based channel to distribute what was otherwise wasted to end consumers that may not otherwise be able to access local seafood. The SDFSA worked with fishers to advocate for regulations that would allow for permitting of a dockside fish market to facilitate connections between fishers and the local restaurants and retailers.

Participant: “fishermen, part of their problem, and this is not San Diego, but it’s all the small fishing communities that have managed to survive, the fishermen are working. They’re out on their boats. They could be 200 miles offshore. It’s not like they can just come in for a Wednesday afternoon two o’clock meeting without impacting their livelihood. They just can’t do it. They’d love to, but they can’t. So having a group like the Alliance who can help go to those meetings, and I mean speak for them, but we can certainly speak from our perspective about how important fishing is to the overall economy, health, wellbeing of San Diego and its food system.”

In this case, the alliance advocated for policy change on behalf of the fishers in order to enable them to continue earning their living.

## Discussion

The next five sections build upon the results of this study and suggest future actions needed to increase the contribution of local seafood to nutritionally vulnerable populations by discussing ways to address the supply chain adaptations, challenges, and enabling factors raised in the studied cases.

### Sourcing Fish that Make Sense for Consumers and Suppliers

Findings from the cases studied demonstrated various ways that the positive deviants successfully adapted and shortened the traditional seafood supply chain, even while relying on a wide array of fish species and sources. Critical to understanding the fish sourcing and distribution process is that each link in the supply chain is costly, especially when the species is unfamiliar. Suppliers generally engage in a search to find buyers who are familiar with their product. For products with reliable demand, these links can be stable (e.g., weekly institutional deliveries) and maintained at a low cost. One species with reliable demand is Alaskan pollock, which is supplied in high volume to SeaShare for distribution to food banks. For products with occasional or highly variable supply (such “bycaught” fish that are caught incidentally while targeting other fish), a costly search is needed with each landing of fish at port. This can be a major obstacle for programs preferentially sourcing underutilized species that tend to have variable supply and little demand. It is difficult to source these underutilized species because fishers do not find them profitable, which in turn signals fishers to avoid catching them. Without consistent fishing effort directed towards underutilized species, their low and variable availability is difficult to market, especially towards large-volume purchasing organizations who need a consistent supply of fish of a specific size and form that their staff are sufficiently familiar with and will utilize.

As illustrated in the case studies, for-profit programs that seek to connect fish with low-income or low-access populations will need to focus on fish that have low value in the mainstream supply chain. It is unlikely that species with existing stable, high-value markets (e.g., salmon, halibut) would be viable selections for budget-based institutional buying programs. However, on the West Coast, these programs do have a considerable opportunity to source from a wide variety of underutilized or bycaught species that are affordable and could be available in nearby harbors. Large volume purchasing organizations like hospitals and schools can make large orders that make handling these species worthwhile for harvesters and processors.

Major shocks to global food systems highlight the importance of identifying local sources of healthy foods. The COVID-19 pandemic has cast harsh light on the vulnerabilities associated with relying on globalized food systems and highlights the critical importance of shortened food supply chains, especially with respect to low-income or food insecure communities (Cappelli & Cini, 2020). The seafood sector has seen massive negative impacts, especially on export-focused production and fish processing. It has also seen large increases in demand for locally sourced seafood, as well as a willingness among private firms to alter their business model to more directly supply consumers (Bennett et al., 2020; Love, Allison et al., 2020). COVID-19 impacts to the supply chain present a challenge and an opportunity for organizations seeking to learn from positive deviant case studies in order to get fish to nutritionally vulnerable consumers. Relying on fish associated with global trade (i.e., local bycaught species associated with export-driven high value target species) may expose consumers to inconsistencies of supply. Increasing consumer interest and demand for local seafood is beneficial for firms seeking to shorten supply chains, but at the same time might push prices for otherwise affordable fish beyond what is viable for organizations seeking to supply low-income consumers. As the pandemic progresses, the fisheries sector will need to shift from short-term coping strategies to long-term adaptation to build resilience in the sector (Love, Allison et al., 2020). Sectors that focus on helping to stabilize food will benefit (i.e., people will rely on them, learn new habits that incorporate them, become aware of locally sourced products); the fisheries sector must harness these shifts in diets to emerge as a staple in the post-pandemic world.

### Sustainability of Seafood Supply

Sustainability concerns could arise as the volume of fish increases to meet demand from large organizations serving low-income or nutritionally vulnerable communities. However, for species caught within the Pacific Coast Groundfish Fishery Management Plan, there is a management safeguard against excessive expansion. Many species have established Total Allowable Catches (TACs) that will be enforced, just as with the current major market species now. For species that are not currently exploited at a level to warrant assessment, mechanisms are in place to ensure they do not become overfished in the event that they experience more fishing effort. In that event, additional monitoring would take place to determine the sustainability of the stock using the same set of rules that were able to successfully rebuild most of previously overexploited stocks (Pacific Fishery Management Council, 2016).

While many of the examples from the case studies related to fish sourced from the ocean, the Clatsop CARFB procured fish from nearby hatcheries. Our results indicate that food banks are already utilizing these “farmed” fish, and the ascendency of aquaculture presents another considerable opportunity for meeting conservation and recreational objectives as well as for contributing to the healthy food system for low-income or nutritionally sensitive populations (Gephart et al., 2020). The growth of the broader aquaculture sector is both well documented and staggering: 15 billion tons of additional fish could be produced, “over 100 times the current global seafood consumption,” all in areas that do not conflict with other uses of the marine environment like marine conservation (Gentry et al., 2017). Aquaculture is oftentimes overlooked as a viable alternative to land-based agriculture. The policy environment in the U.S., for example, has yet to embrace aquaculture; federal spending from 1990 to 2015 was just over US$1 billion while agriculture spending was US$41 billion (Love, Gorski, & Fry, 2017).

### Marketing and Strategies to Improve Consumer Acceptance

Improving the understanding of consumer preferences within low seafood access communities will aid in the identification of preferred, but still underutilized species and in their integration into the healthy food system as an affordable food. For example, Yellowtail rockfish (*Sebastes flavidus*) is broadly distributed along the West Coast and is unfamiliar to many consumers, but has a familiar white flaky texture that is easy to cook. This work could be facilitated by partnering with private-sector organizations such as seafood marketing associations currently trying to improve consumption of regional species. At present, consumer surveys on current and potential seafood preferences are either sparse or rarely conducted. More information is needed about the preferences of nutritionally vulnerable or low-income communities and about the supply chain demands of high-volume organizations. Nonprofits working with regional food banks and local food pantries indicated remarkable flexibility in the utilization of a broad diversity of species either donated by fishing companies and hatcheries or seized by law enforcement. This indicates that local pantries were willing to try species outside of the “big three” and suggests that further sourcing of less typical fish might be possible.

Since consumers are unfamiliar with underutilized species, initiatives must first generate interest in and empower end users to use them. Education was an important component of the strategies used by both Bay2Tray and San Diego Food Systems Alliance to improve understanding of how to cook the fish in institutional settings or at home. Both cases also described innovative recipes that make unfamiliar fish more approachable, like chili made from Opah. Respondents showed that kitchen preparation and familiarity were important enabling conditions for organizations, and we expect that previously unsourced species that are similar in preparation, taste, and appearance to already-consumed products are likely to be more readily adopted in these environments. Messaging is important and may require alternative strategies to the conservation or economic justifications commonly used when advocating for this kind of seafood. A recent study of North American consumers by the Marine Stewardship Council found that consumers make seafood selections based primarily on food safety, freshness, and health benefits rather than the sustainability of the resource or origin (Marine Stewardship Council & Globescan, 2018). While the dominant focus of local seafood advocates is on environmental sustainability, results of this survey indicate a need to increase health and nutrition messaging to better align with the concerns of consumers.

Policy disconnects present obstacles to the sourcing of domestically produced seafood that can readily contribute to the food system. While food policy originates within the USDA, fisheries and aquaculture policies are primarily the purview of National Oceanic and Atmospheric Association, and as such their goals regarding nutrition are oftentimes misaligned (Love, Pinto da Silva, Olson, Fry, & Clay, 2017). For example, because U.S. agricultural policy does not include fisheries and most aquaculture, they are not emphasized in federal food assistance programs like Special Supplemental Nutrition Program for Women, Infants, and Children (WIC) and the National School Lunch program which are housed in the USDA. In U.S. nutrition education programs, seafood is also not well recognized, but it is mentioned alongside other fresh, lean proteins in USDA’s nutrition education program SNAP-Ed as a better alternative to processed meats (USDA Food and Nutrition Service, 2020). Policy guidance is necessary to recognize the potential contributions that fish and shellfish can make alongside other healthy foods already being utilized to meet the goals of these programs.

### Finding Financial Resources are Critical for Program Development

Establishing alternative supply chains requires entities that can play critical roles in processing and distribution. In this regard, reliance on grants was mentioned as critical to all case studies. They were essential to overcoming initial costs to establish an alternative supply chain, and sometimes in covering continuing operational costs. Finding a long-term, financially viable model is paramount, especially for private or public-private partnerships. In the cases studied, scale was consistently mentioned as critical. Oftentimes requisite or desired scaling up requires additional, costly infrastructure. Each node of the supply chain has its costs, and future initiatives need to consider whether they must internalize these costs or work with suitable partners to forward their mission.

While grant acquisition can be undertaken, there are growing opportunities for for-profit firms to seek private investment. Historically, there was limited investment potential for firms focused on nonmonetary or philanthropic objectives, but that has changed with the increases in social impact investment strategies that explicitly seek out firms that advance positive environmental or social outcomes (Pons, Long, & Pomares, 2011). In the U.S., there are a variety of additional public funding mechanisms that help support connecting healthy foods to food insecure populations, often with parallel goals of supporting farmers or economic development. These include USDA-supported farm-to-school programs and the Gus Schumacher Nutrition Incentive Program that incentivizes fruit and vegetable consumption at the point of purchase for low-income consumers participating in the Supplemental Nutrition Assistance Program (SNAP) (USDA, 2019b, 2019a). Parallel programs focusing on fish consumption could take place in state or federal programs to provide potential sources of demand for fish, particularly species that are currently underutilized.

There was little evidence among positive deviant cases studies on the use or feasibility of accessing funding via public-private ventures that connect private and public sector entities. For example, the Healthy Food Financing Initiative was designed to bring grocery stores to communities by funding food retail projects that expand access to healthy foods in underserved areas and create quality employment. Such public-private ventures present another opportunity to support supply of fish and shellfish to low-income populations. For example, a similar financing mechanism could be created to fund efforts connecting fishers or processors to schools, hospitals, or jails.

Philanthropic actions of the fishing industry are buoyed by the ability of regional food banks to source fish, and responses suggest that there need not be tradeoffs for fishing companies between philanthropy and profitability. Some fishing companies reported passing on tax deductions for their contributions, motivated by the community impacts of their donated seafood seen in their community. In U.S. legal contexts, when an organization’s volume and labor costs are high, tax deductions can create incentives for continued operational growth. SeaShare, which works with some of the largest American seafood companies, includes information on its website regarding how companies can qualify for tax deductions under the “Good Samaritan Act,” which currently allows tax write-offs up to twice the cost of the donated product. Growing awareness of the incentives for philanthropy may encourage other large agents in the fishing industry to consider donating product or labor.

### Supporting Champions Throughout the System

Champions were present in every positive deviant case and could arise at any level of influence in the supply chain. Some champions held positions of power in school districts or within the fishing industry; their decisions to increase availability of fish had considerable top-down effect. However, champions were not always the actors with the highest amount of influence in the food system; for example, the willingness of a group of kitchen workers to learn new cooking methods meant they had to spend additional time learning new preparation techniques. When considering the policy process, champions may also become necessary. Many fishers are engaged in *fishery* policy processes, but *food* policy is a different domain, often with different political representatives and therefore the relationships with these politicians are less developed. Champions here may involve bridging organizations with connections to policymakers or with policymakers themselves. Future initiatives should consider that successful interventions in the food system require buy-in from actors throughout the supply chain, regardless of their level of influence, and should actively support their participation with appropriate incentives.

## Conclusion

Taken together, our positive deviant cases provide insight into how underutilized seafood can be directed to low-income and nutritionally vulnerable populations. First, low-income populations traditionally not consuming high volumes of seafood are generally not going to compete within the current supply chain. As a result, a supply of fish not valued by that system must be identified. Positive deviant cases were resourceful in the species they sourced, from species with low market value which are not caught because they are not valued in commercial distribution channels, to high-value species that cannot be sold because they are prohibited bycatch, seized fish, or hatchery products. Second, an alternative, low-cost supply chain can be constructed that keeps revenues high enough to be viable but low enough to be affordable to organizations serving low-income communities. The positive deviant cases cut out the network of fish distributors and traders who disaggregate bundles to the sizes demanded by particular down-stream buyers: the new supply chain is shorter, and deals with large quantity so less effort is required to distribute it. However, constructing this supply channel is itself expensive and time consuming. Here, the energy of a champion of the initiative is key to identifying and developing relationships with others and ensuring work gets done, and grants cover direct costs. The lessons on enabling conditions for the positive deviant case studies and how they overcame challenges provide potential approaches for future initiatives seeking to improve the connection between local seafood and the food system

In future research to better understand the perspectives related to connecting low-income populations with fish, interviews should be directed towards the fishers, hatchery managers, and broadline distributors that were not included in this research. Interviewing potential end consumers who are or could be the beneficiaries of these arrangements would also be critical to the development of pilot programs. With an improved understanding of the challenges and conditions enabling successful distribution of fish from local markets *throughout* the supply chain to nutritionally vulnerable populations, work can begin to implement and evaluate pilot programs in organizations and regions where they do not exist. In addition to the types of organizations that led the initiatives studied in these cases, others may be as or better suited to serve as champions for this work, including religious groups or cultural centers. Likewise, efforts like those studied may be successful in other food service and retail settings and mechanisms, such as correctional facility food services or direct-to-consumer approaches. Promising initiatives can then scale their impact to improve the flow of local seafood to nutritionally vulnerable people who access food through schools, food banks, hospitals, and other food service settings along the coast.

## Figures and Tables

**Table 1.** Description of Interviewees

Organizations represented	# of interviewees
Nonprofit food rescue/emergency food	5
Schools	1
Hospitals	1
Fish-related business/entrepreneur	2
Community food coalition	1

Note There are 10 organizations represented, but only nine interviewees because one interviewee represented two organizations.

**Table 2.** Characteristics of U.S. West Coast Positive Deviant Cases Connecting Underutilized Fish to Food Insecure and Nutritionally Vulnerable Populations^a^

*Case*	Location	Description	How it bridged the supply chain	Scale	Food environment
**SeaShare**	Bainbridge (WA)	Established in 1994, this nationwide nonprofit directs donated fish harvested in the North Pacific from seafood companies across the U.S. to food banks. It connects the nation’s largest network of food banks, Feeding America, to some of the largest domestic seafood companies based in the Pacific Northwest. SeaShare’s role is to organize the supply chain to facilitate these donations.	Developed a pathway for major fisheries to contribute to emergency foods, unlike agriculture; by establishing relationships to create connection in existing supply chain.	Nationwide	Regional food banks and food pantries
**Clatsop Community Action Regional Food Bank (Clatsop CARFB)**	Warrenton (OR)	Starting in 2012, Clatsop county’s largest regional food bank began sourcing and processing local seafood on a donation basis for its partner food pantries. It later outsourced custom processing to a nearby seafood processing plant. Once processed, the food bank picks up the fish and integrates it into its existing delivery to local food pantries.	Identified a supply of otherwise wasted seafood across a variety of sectors; used transportation to bridge gap between suppliers, processors and food pantries.	Single county	Food pantries
**Bay2Tray**	Moss Landing (CA)	This program was started in 2014 as part of Real Good Fish, a for-profit direct-to-consumer seafood firm based in Central California. Bay2Tray uses the firm’s vertically integrated approach to source fish from fishers for direct-delivery to schools. Once the fish is purchased, it is processed, portioned into school servings, packed for delivery, and transported to schools where the school kitchens prepare the fish for service in the cafeteria.	Identified supply chain between local fishers and schools; by intervening in supply chain by purchasing, processing, marketing and distributing fish to schools.	Multiple counties in single region	School districts, and on to participating school lunch programs
**San Diego Food Systems Alliance & Seafood Working Group (SDFSA)**	San Diego (CA)	Launched in 2012, this collaborative works to support the sustainability and economic strength of the local food system. It convenes a seafood working group that supports connecting hospitals and schools to the local fishing industry and encourages distributors to source from local harbors instead of imported or nonlocal commodity species. The Alliance also works with fishers to advocate for regulations that allowed for permitting of a dockside fish market.	Kitchen workers had relatives or friends in fisheries, desire to support them and support local seafood industry.	Single county	Individual restaurants, hospitals, schools

a Information in this table was synthesized from initiative websites and from local news coverage. SeaShare’s website is https://www.seashare.org/. CCARFB website is https://ccaservices.org/food/food-pantries/, information was also gathered on Clatsop CARFB from the Astorian, a local news outlet (Heffernan, 2017). Bay2Tray’s website is https://www.realgoodfish.com/bay2tray, information was also gathered from food media outlet Civil Eats (Guth, 2016). The SD Food Systems Alliance Seafood Working Group website is https://www.sdfsa.org/sustainable-local-seafood, information was also gathered from Asparagus Magazine (Kwon, 2018).

**Table 3.** Enabling Conditions of Positive Deviant Cases

Enabling conditions	Funding and Financial Incentives	Champions	Cultural Connection with Fishing Heritage
SeaShare	Grants; federal tax incentives enabled seafood company donations.	Board members representing heads of donating seafood companies.	Pantries located in neighborhoods with communities from cultures that saw high demand
Clatsop CARFB	Grants; labor and packaging donated by large, local processor.	Processor who donated fish cutting labor and packaging, after volume of fish became too large for food bank to complete in-house.	Proximity to local harbor meant many food bank and pantry workers were familiar with seafood processing and handling.
Bay2Tray	Grants.	Nutrition directors and superintendents willing to innovate; kitchen workers willing to learn to cook from scratch; high school student groups supported local food sourcing.	Kitchen workers had relatives or friends in fisheries, desire to support them.
SDFSA	Broader program funded by grants and donations.	Chefs at institutional kitchens willing to create and test new recipes with less familiar fish; members of the SDFSA Seafood working group advocating for advantageous regulations and local fish.	Desire to support local food producers.

## Box 1. Typical Seafood Supply Chain

Fishers participating in capture fisheries seek to harvest wild fish or shellfish that have strong markets. These “target species” are purchased by “first buyers” who are sometimes also processors who turn it into useable product, such as fillets. Once the fish are landed, some are exported (depending on the species) but those remaining in domestic markets are bought by wholesalers. Large-scale wholesalers distribute to broader geographies and major markets, potentially nationwide. Seafood produced from aquaculture often enters West Coast markets at this point, as much of it is produced internationally and imported through large-scale wholesaler operations. Small-scale wholesalers tend to distribute to more local markets. Distributors then sell the product to consumer-facing institutions (e.g., hospitals) or retail (e.g., supermarkets). Waste may occur at any node in the supply chain. This may include fish discarded by fishers at sea because they do not have market value (this is often regulated), processing byproducts from the creation of fillets, or spoilage. Spoilage may occur throughout distribution, or when stored by consumers. Much of the global volume of seafood passes through large businesses that own fishing vessels, processing plants, and transportation, and sell to larger-scale distributors (Österblom et al., 2015).

## References

[R1] Bennett NJ, Finkbeiner EM, Ban NC, Belhabib D, Jupiter SD, Kittinger JN, Mangubhai S, Scholtens J, Gill D, & Christie P (2020). The COVID-19 pandemic, small-scale fisheries and coastal fishing communities. Coastal Management, 48(4), 1–11. 10.1080/08920753.2020.1766937

[R2] Bogard JR, Thilsted SH, Marks GC, Wahab MA, Hossain MAR, Jakobsen J, & Stangoulis J (2015). Nutrient composition of important fish species in Bangladesh and potential contribution to recommended nutrient intakes. Journal of Food Composition and Analysis, 42, 120–133. 10.1016/j.jfca.2015.03.002

[R3] Cappelli A, & Cini E (2020). Will the COVID-19 pandemic make us reconsider the relevance of short food supply chains and local productions? In Trends in Food Science & Technology (Vol. 99, pp. 566–567). Elsevier Ltd. 10.1016/j.tifs.2020.03.041 32288230 PMC7138154

[R4] Cvitanovic C, & Hobday AJ (2018). Building optimism at the environmental science-policy-practice interface through the study of bright spots. Nature Communications, 9, 3466. 10.1038/s41467-018-05977-w PMC6113225 30154434

[R5] Errend M, Gilden J, Harley A, Morrison W, Pfeiffer L, Russell S, & Seger J (2017). West coast groundfish trawl catch share program five-year review (Issue 12). Retrieved from https://www.pcouncil.org/documents/2017/11/agenda-item-f-2-attachment.pdf/

[R6] Farmery AK, van Putten IE, Phillipov M, & McIlgorm A (2020). Are media messages to consume more underutilized seafood species reliable? Fish and Fisheries, 21(4), 844–855. 10.1111/faf.12467

[R7] Gentry RR, Froehlich HE, Grimm D, Kareiva P, Parke M, Rust M, Gaines SD, & Halpern BS (2017). Mapping the global potential for marine aquaculture. Nature Ecology & Evolution, 1(9), 1317–1324. 10.1038/s41559-017-0257-9 29046547

[R8] Gephart JA, Golden CD, Asche F, Belton B, Brugere C, Froehlich HE, Fry JP, … & Allison EH (2020). Scenarios for global aquaculture and its role in human nutrition. Reviews in Fisheries Science & Aquaculture, 1–17. 10.1080/23308249.2020.1782342

[R9] Guth A (2016, 11 7). “Boat-to-school” programs source fresh seafood for students. Civil Eats Retrieved from https://civileats.com/2016/11/07/boat-to-school-programs-source-fresh-seafood-for-students/

[R10] Heffernan J (2017, 10 11). Fish on the menu. The Astorian. Retrieved from https://www.dailyastorian.com/news/local/fish-on-the-menu/article_3292acf3-69b2-5c50-9d1b-b044dcde7913.html

[R11] Hicks CC, Cohen PJ, Graham NAJ, Nash KL, Allison EH, D’Lima C, Mills DJ, … & MacNeil MA (2019). Harnessing global fisheries to tackle micronutrient deficiencies. Nature, 574, 95–98. 10.1038/s41586-019-1592-6 31554969

[R12] Jahns L, Raatz SK, Johnson LK, Kranz S, Silverstein JT, & Picklo MJ (2014). Intake of seafood in the US varies by age, income, and education level but not by race-ethnicity. Nutrients, 6(12), 6060–6075. 10.3390/nu6126060 25533013 PMC4277015

[R13] Kwon S (2018, 2 28). San Diego-area chef works to turn the tide for local seafood. [Blog post]. Asparagus Magazine. Retrieved from https://medium.com/asparagus-magazine/san-diego-area-chef-works-to-turn-the-tide-for-local-seafood-69b4ecf0d0b9

[R14] Larson NI, Story MT, & Nelson MC (2009). Neighborhood environments: Disparities in access to healthy foods in the U.S. American Journal of Preventive Medicine, 36(1), 74–81.e10. 10.1016/j.amepre.2008.09.025 18977112

[R15] Lewis NM, Widga AC, Buck JS, & Frederick AM (1996). Survey of omega-3 fatty acids in diets of midwest low-income pregnant women. Journal of Agromedicine, 2(4), 49–58. 10.1300/J096v02n04_05

[R16] Loring PA, Gerlach SC, & Harrison HL (2013). Seafood as local food: Food security and locally caught seafood on Alaska’s Kenai Peninsula. Journal of Agriculture, Food Systems, and Community Development, 3(3), 13–30. 10.5304/jafscd.2013.033.006

[R17] Love DC, Allison EH, Asche F, Belton B, Cottrel RS, Froehlich HE, … Zhang W (2020). Emerging COVID-19 impacts, responses, and lessons for building resilience in the seafood system. OSF Preprints, 1–22. 10.31235/osf.io/x8aew PMC8417121 34513582

[R18] Love DC, Asche F, Conrad Z, Young R, Harding J, Nussbaumer EM, Thorne-Lyman AL, & Neff R (2020). Food sources and expenditures for seafood in the United States. Nutrients, 12(6), 1810. 10.3390/nu12061810 PMC7353403 32560513

[R19] Love DC, Gorski I, & Fry JP (2017). An analysis of nearly one billion dollars of aquaculture grants made by the U.S. federal government from 1990 to 2015. Journal of the World Aquaculture Society, 48(5). 10.1111/jwas.12425

[R20] Love DC, Pinto da Silva P, Olson J, Fry JP, & Clay PM (2017). Fisheries, food, and health in the USA: The importance of aligning fisheries and health policies. Agriculture & Food Security, 6(16), 1–15. 10.1186/s40066-017-0093-9

[R21] Marine Stewardship Council, & Globescan. (2018). Understanding & activating seafood consumers–North America [Slideshow] Retrieved from https://msc20-live-cd2.msc.org/docs/default-source/default-document-library/for-business/rise-of-the-conscious-food-consumer---north-america-webinar-slides.pdf?sfvrsn=f32d573_4

[R22] Marty L, Dubois C, Gaubard MS, Maidon A, Lesturgeon A, Gaigi H, & Darmon N (2015). Higher nutritional quality at no additional cost among low-income households: insights from food purchases of “positive deviants.” The American Journal of Clinical Nutrition, 102(1), 190–198. 10.3945/ajcn.114.104380 26016868

[R23] Miller H (2015, 1 13). Huge hatchery returns help feed the hungry. Statesman Journal. Retrieved from https://www.statesmanjournal.com/story/travel/outdoors/hunting-fishing/2015/01/13/huge-hatchery-returns-help-feed-hungry/21688357/

[R24] National Oceanic and Atmospheric Administration Fisheries. (2019). Status of stocks 2018: Annual report to Congress on the status of U.S. fisheries Retrieved from https://www.fisheries.noaa.gov/feature-story/status-stocks-2018

[R25] Österblom H, Jouffray J-B, Folke C, Crona B, Troell M, Merrie A, & Rockström J (2015). Transnational corporations as “keystone actors” in marine ecosystems. PloS One, 10(5), e0127533. 10.1371/journal.pone.0127533 26017777 PMC4446349

[R26] Pacific Fishery Management Council. (2016). Pacific coast groundfish fishery management plan: For the California, Oregon, and Washington groundfish fishery.Retrieved from https://nrm.dfg.ca.gov/FileHandler.ashx?DocumentID=161281&inline

[R27] Pascale RT, & Sternin J (2005). Your company’s secret change agents. Harvard Business Review. Retrieved from https://hbr.org/2005/05/your-companys-secret-change-agents 15929405

[R28] Pérez-Roda MA, Gilman E, Huntington T, Kennelly SJ, Suuronen P, Chaloupka M, & Medley PAH (2019). A third assessment of global marine fisheries discards (FAO Fisheries and Aquaculture Technical Paper No. 633) Retrieved from FAO website: https://www.fao.org/

[R29] Pons E, Long M-A, & Pomares R (2011). Promoting sustainable food systems through impact investing. Retrieved from The Springcreek Foundation website: http://www.thespringcreekfoundation.org/images/download/tsf_Promoting_Sustainable_Food_Systems_1212.pdf

[R30] Savoca MS, Brodie S, Welch H, Hoover A, Benaka LR, Bograd SJ, & Hazen EL (2020). Comprehensive bycatch assessment in US fisheries for prioritizing management. Nature Sustainability, 3, 472–480. 10.1038/s41893-020-0506-9

[R31] Sepez J, Lazrus H, Milne N, Package C, Russell S, Grant K, Lewis R, Primo J, Springer E, & Styles M (2007). Community profiles for west coast and north pacific fisheries, Washington, Oregon, California, and other U.S. States (NOAA Technical Memorandum NMFS-NWFSC-85) Retrieved from NOAA Fisheries website: http://www.nwfsc.noaa.gov

[R32] Shannon KL, Kim BF, McKenzie SE, & Lawrence RS (2015). Food system policy, public health, and human rights in the United States. Annual Review of Public Health, 36(1), 151–173. 10.1146/annurev-publhealth-031914-122621 25785888

[R33] Talley TS, Warde H, & Venuti N (2016). Local seafood availability in San Diego, California seafood markets. Future of Food: Journal on Food, Agriculture and Society, 4(2), 40–49. Retrieved from https://www.thefutureoffoodjournal.com/index.php/FOFJ/article/view/92

[R34] U.S. Center for Disease Control, & National Center for Environmental Health. (2012). Second national report on biochemical indicators of diet and nutrition in the U.S. population https://www.cdc.gov/nutritionreport/pdf/nutrition_book_complete508_final.pdf

[R35] U.S. Department of Agriculture [USDA]. (2019a). Farm to school grant program. Retrieved from USDA-FNS website: https://www.fns.usda.gov/cfs/farm-school-grant-program

[R36] USDA. (2019b). Gus Schumacher Nutrition Incentive Program (formerly FINI). Retrieved from National Institute of Food and Agriculture website: https://nifa.usda.gov/program/gus-schumacher-nutrition-incentive-grant-program

[R37] USDA Food and Nutrition Service. (2020). SNAP-Ed toolkit. https://snapedtoolkit.org/

[R38] U.S. Department of Health and Human Services, & USDA. (2015). 2015–2020 Dietary Guidelines for Americans (8th Edition). Retrieved from https://health.gov/our-work/food-nutrition/2015-2020-dietary-guidelines/guidelines/

[R39] U.S. Environmental Protection Agency. (2014). Estimated fish consumption rates for the U.S. population and selected subpopulations (NHANES 2003–2010) (Issue 4). https://www.epa.gov/sites/production/files/2015-01/documents/fish-consumption-rates-2014.pdf

